# Two Cases of Restlessness—I

**Published:** 1908-03-21

**Authors:** Charles Mercier

**Affiliations:** Physician for Mental Diseases to Charing Cross Hospital.


### Mental Diseases.

### TWO CASES OF RESTLESSNESS.—I.

By CHARLES MERCIER, M.D., F.R.C.P., Physician for Mental Diseases to Charing Cross Hospital.

Mere restlessness is not a matter for which physicians are often consulted. It is on the face of it an unimportant malady; but when it exists in sufficient intensity to form the subject of complaint, and to induce the sufferer to seek advice, it is usually found to be the superficial indication of a grave underlying condition as in the cases hereunder described.

A. B., a member of a learned profession, aged 25, was brought to see me on account of "extreme restlessness." He could not settle to anything. When reading or at work he would, after a short time, throw down his book and go out for half an hour. Then he would come in, and, after a short spell of work, out he would go again. In the middle of a meal he would throw down his knife and fork, and walk out of the room and out of the house. If he were playing billiards or whist, he would similarly lay down his cue or his cards, and walk out of the room and out of the house without explanation or apology. He never kept to any occupation for more than ten minutes, and spent much of his time in wandering about the country lanes near his house. The only other odd things he did were that he sometimes lost his way in places that he knew well; and he had several times called at the houses of people he did not know, walked in when the door was opened, stared about him, and gone away without saying a word. About twenty months before he came to me he had had a love affair, which had not resulted favourably, and had been followed by a "nervous breakdown," the details of which were obscure, but which was of sufficient gravity to alarm his parents, and induce them to take him abroad for some time. He talked and argued reasonably, and when asked to explain his restlessness merely said he could not concentrate his thoughts.

This was the account I received from his medical attendant, and I then interviewed the patient, a well made and well set-up young man, with a good head, an intelligent face, and a reserved and suspicious expression. When the description above given was put to him, he admitted its correctness, but declared that he could not account for his conduct in any way except that he was abnormally restless, could not concentrate his thoughts on what he was doing, and felt impelled to get up and wander about.

This was very unenlightening; but it was clear that the condition, whatever it was, dated from the "nervous breakdown," and therefore probably resulted from it, and was in some way connected with its cause. I therefore questioned him about his love affair. It then appeared that his acquaintance with the lady had been brief and slight, that he had not actually been refused, for he had never proposed to her, but that she made so great an impression upon him that he thought of her perpetually, and often, when he saw a lady at a distance, thought it was she, only to be undeceived when he came close up to the object of his attention. He could not help thinking that he should see her again; and, considering that they both moved in much the same society, there seemed nothing unreasonable in this. When asked point-blank why he was in the habit of leaving abruptly what he was doing, and going out of the room and out of the house, he said he had a good reason, but declined to give it. At the same time he covered his mouth with his hand, averted his eyes, and smiled, a smile that was a peculiar mingling of sheepishness and knowingness. This gave me a clue. The true state of affairs flashed upon me immediately. I said. "*You go to meet the lady.*" He looked up in astonishment, and, taken aback, admitted that this was the case. "And when you call at houses of people you do not know, you go with the expectation of finding her there." Again he admitted that the surmise was true; and then he made a clean breast of the whole affair. His mind was possessed with the image of his adored. He was always thinking of her, and the feeling frequently came over him that if he were to go out he should meet her. He has an urgent, unaccountable feeling that she is somewhere in the neighbourhood, and that he must go out and find her. On further cross-examination I elicited the surprising intelligence that the lady he expects to meet is not the lady he was in love with. It is not any particular lady that he has ever known. The feeling is that there is some lady in the neighbourhood with whom he is *en rapport*, and whom he urgently desires to meet. He is not sure that it is but one lady. There may be more. He has on more than one occasion been passed by a lady driving by in a carriage, and it has flashed upon him that this was the lady of his search. What made him think so was that the sight of her was followed by a feeling of elation and exaltation. He felt indescribably strong and capable. It is the hope of experiencing this feeling again that impels him to go out and search for the lady whenever the conviction comes over him, as it so often does, that she is somewhere at hand and could be found by sufficient search. Until he is successful he will never, he says, be able to settle down to anything.

The state of things here revealed opens out a number of vistas, any one of which would be most interesting to follow. Three hundred years ago he would have been regarded, would probably have regarded himself, as the victim of witchcraft, and his malady might have been fatal to half a score of old women—or young women, for the matter of that. He is undoubtedly of unsound mind, but it is impossible to classify his malady under any known and described form of insanity. No doubt the followers of Kraepelin would regard it as a case of "dementia precox," but as this title is applied indiscriminately to every case of insanity in the young—not excluding the middle-aged and the old—it does not help us very much. Evidently, however, it is a very grave and deep-rooted mental disorder, strongly affecting conduct, and chronic in its nature, and the prognosis given was accordingly bad.

